# Trading Network Predicts Stock Price

**DOI:** 10.1038/srep03711

**Published:** 2014-01-16

**Authors:** Xiao-Qian Sun, Hua-Wei Shen, Xue-Qi Cheng

**Affiliations:** 1 Institute of Computing Technology, Chinese Academy of Sciences, No.6 Kexueyuan South Road Zhongguancun, Haidian District, 100190, Beijing, China

## Abstract

Stock price prediction is an important and challenging problem for studying financial markets. Existing studies are mainly based on the time series of stock price or the operation performance of listed company. In this paper, we propose to predict stock price based on investors' trading behavior. For each stock, we characterize the daily trading relationship among its investors using a trading network. We then classify the nodes of trading network into three roles according to their connectivity pattern. Strong Granger causality is found between stock price and trading relationship indices, i.e., the fraction of trading relationship among nodes with different roles. We further predict stock price by incorporating these trading relationship indices into a neural network based on time series of stock price. Experimental results on 51 stocks in two Chinese Stock Exchanges demonstrate the accuracy of stock price prediction is significantly improved by the inclusion of trading relationship indices.

Stock price prediction is a fundamental problem for financial market analysis and has attracted much research attention from both academia and industrial sectors. Stock price prediction aims to determine the future value of a company stock or other financial derivatives. Early research is mostly based on efficient-market hypothesis and random walk theory^1^,^2^,^3^,^4^,^5^. Efficient-market hypothesis states that stock price is a full reflection of all relevant information. This implies that current stock price is basically determined by currently available information rather than past prices. As a result, stock price follows a random walk pattern and thus cannot be predicted with more than 50 percent accuracy^6^.

Is stock market really unpredictable? Actually, efficient-market hypothesis is challenged ever since it is proposed. Driven by profit or other incentives, many investors, stock analysts, stock brokers, and even scientists made their efforts to predict stock price. Generally, those efforts aim to find some clues or indictors for future stock price. Early practitioners are concerned with listed companies raising capital via stocks and predict stock price by assessing the operation performance of listed companies. Alternatively, some studies predict future stock price by leveraging historical stock price and exploiting long-range correlation of the time series of stock price^7^,^8^.

In recent years, statistical models are adopted to construct prediction tools on various kinds of stock market^18^,^19^,^20^,^21^. Among them, neural network gains its success as a promising tool for analyzing and predicting financial time series^6^,^22^. Qian et al. predicted the Dow Jones Industrial Average index by using Hurst exponent to select a period with great predictability and they got a prediction accuracy of 65 percent^8^. Bollen et al. argued that some predictive indicators could be extracted from online website although stock price is affected by certain unpredicted news or exogenous shock. They proposed to use the collective mood states derived from Twitter to predict the value of the Dow Jones Industrial Average with the accuracy up to 87.6 percent^9^. Preis et al. introduced a method to identify online precursors for stock market movements, using trading strategies based on search volume data provided by Google Trends. By analyzing Google search volume for 98 terms of varying financial relevance, they found that the increases in search volume for financially relevant search terms tend to precede large losses in financial markets^10^,^11^. Bordino et al. find the queries volume in web search related to a particular stock and the daily exchanges volume over the same stock have a time-lagged cross-correlation^12^.

However, existing studies mainly focus on the prediction of composite index. It is still an open problem to predict the price of individual stock. Basically, the price of an individual stock reflects the collective judgment to the value of stock and it is thus determined by the supply and demand of stock if without any interference. More specifically, the price of a stock depends on the trading behavior of its investors. In stock market, large market movement arises from trading behavior of large investors^13^. If a large investor sells its holdings of a stock, the sale usually corresponds to a daily turnover of trading volume and stock price. This is because big sale invokes a great number of traders buying the shares. In general, investors collect all the relevant information which they could get and then make a decision to buy or sell a certain stock. The decision of an individual investor may be based on incomplete information, but the collective behavior of all investors could remedy the lack of information and finally determine the price of a stock. As a conclusion, the study of trading behavior of investors could help us understand the dynamics of stock market and predict future stock price^14^,^15^,^16^,^17^.

In this paper, we use stock transaction data to analyze the potential power of trading behavior at predicting price of individual stock. For each stock, we construct a stock trading network to characterize the daily trading relationship among its investors. Then we classify the nodes of trading network into three categories, i.e., hub nodes, periphery nodes, and connector nodes, according to their roles in maintaining connectivity of trading network^23^. Accordingly, trading relationships in trading network are grouped into 9 types in terms of the roles of its adjacent nodes. Using Granger causality analysis, we find that stock price is strongly correlated with trading relationship indices, i.e., the fraction of trading relationship among different kinds of nodes. Furthermore, by combining these trading relationship indices together with time series of stock price, we propose a stock price prediction model based on feed forward neural network. Experimental results on 51 stocks in two Chinese Stock Exchanges demonstrate that the accuracy of stock price prediction is significantly improved by the inclusion of trading relationship indices.

## Results

**Figure 1:**
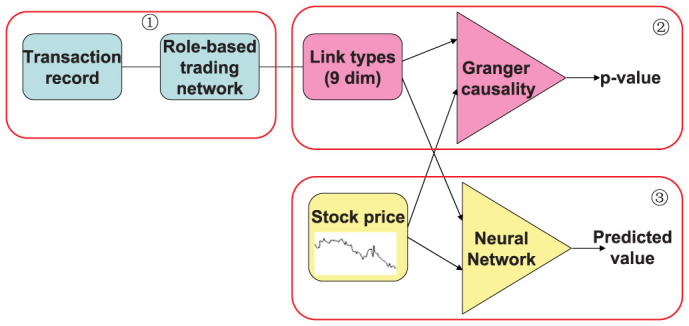
Diagram of methodology: (1) construction of role–based trading network, (2) Granger Causality analysis, (3) stock price prediction based on neural networks.

Our analysis is conducted on transaction data (see the Methods section). The analysis consists of three stages, as shown in Fig. 1. The first stage is data preparation. For each stock, we construct daily trading network based on its daily transaction data and give a role-based description of trading network (see the Methods section). In this way, we obtain 9 time series respectively depicting the evolution of link type fraction over time. We also extract the time series of daily closing price of stock. In total, we have 10 time series for each stock. In the second stage, we conduct Granger causality analysis to investigate whether the fraction of links is predictive factors of stock price. Finally, we predict stock price by deploying a three-layered feed forward neural network with the above 10 time series as inputs. We also check the effect of the fraction of links by comparing the prediction accuracy of neural networks with and without these factors.

### Role-based description of trading network

Traders in stock market decide when to buy or sell based on all information they could get. Stock price continuously changes as traders buy or sell their shares. Although it is hard to fully capture all the information that underlies traders' decision, the outcome of their decision and the trading relationship among them are precisely reflected by stock trading network. Intuitively, traders with various volumes of shares behave differently and play different roles at affecting stock price. For example, traders with large shares prefer position trading whereas traders will less shares usually pursue swing trading or even day trading.

**Figure 2:**
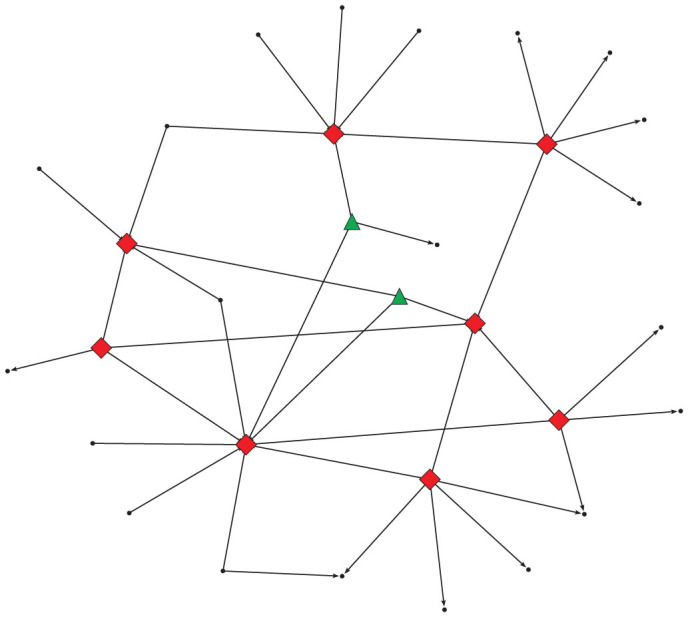
Role-based description of a trading network for a stock on one trading day. Hubs, periphery nodes and connector nodes are respectively depicted by diamonds, circles and triangles.

In stock trading network, nodes may exhibit different connecting patterns or play different roles at maintaining the connectivity of trading network. As shown in Fig. 2, links in stock trading network are dominated by a handful of hubs, which are surrounded by a huge number of periphery nodes. Meanwhile, several broker-like nodes connect hubs and periphery nodes. In sum, stock trading network is made up of nodes with different roles at maintaining the connectivity of trading network. We denote these three types of nodes by H(hub), C(connector) and P(periphery).

We further classify links in trading network into 9 categories, according to the roles of its two adjacent nodes. For each link, the outgoing node from which the link departs has three potential roles and the incoming node to which the link points also has three possible roles. For convenience, we denote the 9 link categories as P-P, P-C, P-H, C-P, C-C, C-H, H-P, H-C, and H-H. The first letter denotes the role of outgoing node and the second letter denotes the role of incoming node. In this way, we obtain a role-based description of trading network with nodes and links being assigned different roles.

**Figure 3:**
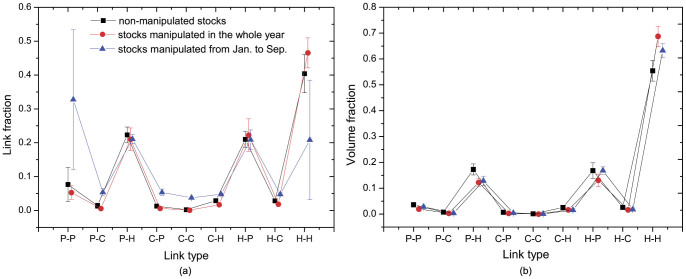
Distribution of link types in trading networks: (a)link fraction, (b)volume fraction.

The role-based description of trading network could provide critical indictors for understanding trading behavior and predicting stock prices. As an illustration, we now show that the distribution of links over 9 categories could differentiate manipulated stocks from non-manipulated stocks, and distinguish manipulated stocks in different manipulation period. Figure 3(a) depicts the link distribution and volume distribution of 9 kinds of links for 43 non-manipulated stocks, 5 manipulated stocks with a whole-year manipulation period, and 3 manipulated stocks in their late manipulation period spanning from January to September in 2004. As shown in Fig. 3(a), remarkable differences are observed in the fraction of link types H-H and P-P. The fraction of H-H links for manipulated stocks in late manipulation period is significantly lower than that of the other two kinds of stocks. The fraction of H-H links in manipulated stocks with a whole-year manipulation period is marginally higher than that in non-manipulated stocks. For the case of P-P links, the results are opposite. This captures and confirms the following intuition: for manipulated stocks in early manipulation period, manipulators with large shares frequently engage in fraudulent trading among themselves to create an image of active market. As a result, the fraction of H-H links is higher than non-manipulated stocks. For manipulated stocks in late manipulation period, manipulators attempt to reduce their shares gradually. As a result, the fraction of H-H links becomes lower than non-manipulated stocks. This could also explain the difference in the fraction of P-P links. Fig. 3(b) shows that the volume fraction has the same trend as the link fraction. According to volume fraction, the top-3 link types are H-H, H-P, P-H, reflecting the dominance of hub nodes (traders with high stock share) at trading behavior.

### Role-based stock prediction

We now check the predictive power of role-based link types. Given a stock, we calculate the fraction of 9 types of links and then use Granger causality test to check whether each link type is predictive of stock price. Table 1 shows the p-values of Granger causality test with respect to nine link types and lag days from 1 day to 7 days. Link type P-H has significant Granger causality relation with stock price for 2 lag days (*p* – *value* < 0.05) and 3 lag days (*p* – *value* < 0.1). Link type C-H shows significant Granger causality relation with stock price for 1 to 3 lag days (*p* – *value* < 0.1). The other seven link types do not exhibit significant Granger causality relations with stock price. These results partly reject the hypothesis that link types do not Granger-cause the change of stock price. In addition, by checking the time series of link type and stock price, we find that the time series of link types P-H and C-H are 3 days earlier than the time series of stock price change. On the contrary, for their opposite link types H-P and H-C, no significant Granger causality is observed. This implies that big bid orders could affect future stock prices while big ask orders could not.

Granger causality analysis is based on linear regression model, which could not uncover the non-linear relation between link types and stock price. Therefore, we further develop a neural network model for stock price prediction. According to the result of Granger causality test, the two-link types P-H and C-H exhibit significant Granger causality to stock price. Thereafter, we focus on evaluating the predictive power of these two link types. The evaluation is conducted by developing neural networks with the following three kinds of inputs: 
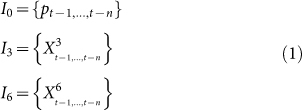
where *p_t_*_−*i*_(1 ≤ *i* ≤ *n*) is the stock price in the day leading the day t for *i* days, 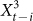 is the fraction of link P-H in the day leading the day t for *i* days, and 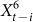 is the fraction of link C-H in the day leading the day *t*. The results are shown in Table 2. We can draw several conclusions from these results. First, the prediction performance of 3 days later is better than that of 2 days later. Second, there is no remarkable difference between the performance of only using a single attribute and only using historical price.

We further evaluate the prediction performance by combining the time series of link type P-H or C-H with the time series of stock price. Meanwhile, to study whether other link type could provide some improvements on prediction accuracy, we also consider the other 7 link types. In this way, for link type P-H, the inputs to neural networks are 
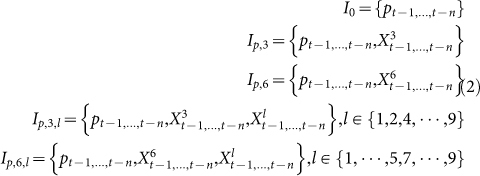
As shown in Table 3, MAPE is reduced by combining link type P-H with time series of stock price while the performance of prediction deteriorates when combining link type C-H with time series of stock price. Moreover, by incorporating link type P-P (i.e., *X*^1^) with link type P-H, the prediction performance is significantly improved, with MAPE reduced from 4.50 to 3.95 and accuracy increased from 56.7% to 61.7%.

Finally, to clarify the predictive power of link types, we remove the time series of stock price from the inputs of neural networks. This means that we only leverage the information of link types to predict stock price. For these cases, the inputs of neural networks are 
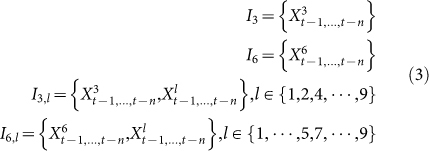
By comparing Table 4 with Table 3, we can see that MAPE is greatly reduced when we do not leverage the past information of stock price. It seems that the past information of stock price is harmful (at least not helpful) for predicting future stock price. However, the accuracy for rise or fall prediction decreases for most combination. The best prediction accuracy and MAPE are obtained when we combine link type C-H (i.e., *X*^6^) with H-P (i.e., *X*^7^). Note that the best performance is obtained by incorporating the link type H-P which has no Granger causality relation with stock price. These results show that link type predicts stock prices in a certain non-linear way.

**Figure 4:**
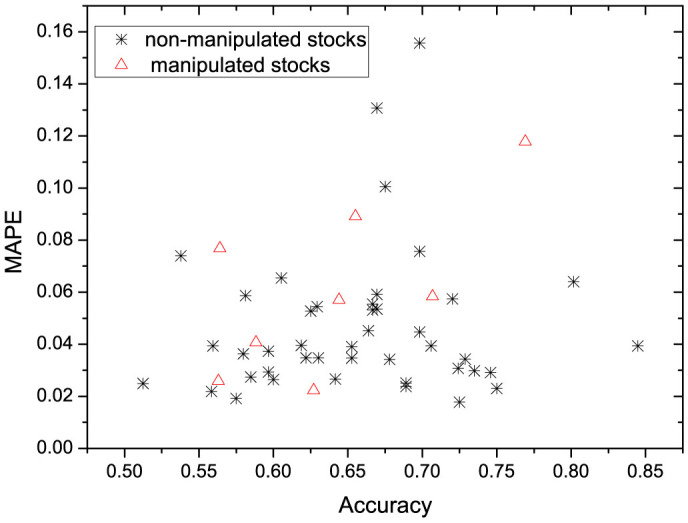
Scatter plot of MAPE and accuracy on 43 non-manipulated stocks and 8 manipulated stocks.

We perform the method on our data set. Figure 4 depicts the scatter plot of the prediction accuracy and MAPE for all 51 stocks, 43 non-manipulated stocks and 8 manipulated stocks. We can see that MAPE is below 10% for 92% of these stocks. The accuracy of prediction is above 60% on about 74% of all non-manipulated stocks. For manipulated stocks, the prediction accuracy is slightly lower. One possible reason is that the stock price is manipulated by colluded traders and becomes less predictable using only past stock price.

## Discussion

We investigated the problem of stock price prediction for individual stock rather than predicting global indices of stock market as done by traditional studies. Our study is based on transaction data, which record the executed transaction generated by electronic trading system in stock exchange. This kind of data provides us an effective way to grasp the trading relationship among investors and provide us a potential way to learn the trading behavior of investors. Based on transaction data, we construct stock trading network for each stock. Taking stock trading network as a proxy, we study whether trading behavior could predict the change of stock price.

We classified nodes in trading networks into three categories according to their roles at maintaining the connectivity of trading network. Then, links are classified into 9 types which characterize the trading behavior of different kinds of investors. Using Granger causality test, we find that some types of links (e.g., P-H and C-H) are predictive for the change of stock price while other are not. This founding indicates that big bid orders could affect future stock prices while big ask orders could not. The predictive power of link types reach its best when predicting the stock price in the future 2 to 3 days. To further demonstrate the effectiveness of link types at predicting stock prices, we deployed a feed forward neural network which takes time series of stock price and time series of link types as input. Results showed that the inclusion of link type could significantly improve the accuracy of stock price prediction.

Our goal is to study the effects of incorporating information from trading network on the prediction of stock price. Thus, we do not try to propose an optimal model for stock price, by carefully studying technical problems such as how to define the role of nodes, how to determine the size of training set and how to incorporate more link properties. We look forward to seeing such kind of work for improving the prediction of stock price. Furthermore, much future work is desired by considering more factors, such as holding time and trading volume. In addition, social network services provide valuable tools to gather and learn the opinions about stock market and mood of investors. As future work, we will devote to combining these kinds of information with the information from trading network to further improve the prediction of stock price.

## Methods

### Stock trading network

The data used in this paper are transaction data of stocks traded on Shanghai Stock Exchange and Shenzhen Stock Exchange in 2004. Transaction data record all executed orders. In total, the data consist of 51 stocks with 14,423,175 transaction entries, involving 3,926,805 unique trader accounts. Each entry records the date and time of transaction, a unique transaction number, the buyer id, the seller id, the volume and price. Among all these 51 stocks, eight stocks had been manipulated by some investors via trade-based manipulation, as revealed by China Securities Regulatory Commission (CSRC). In addition, among the eight manipulated stocks, the manipulation period of five stocks persists through the whole year of 2004. For the other three manipulated stocks, the manipulation period covered by our data is from Jan. 2004 to Sep. 2004, indicating that these stocks are in the late manipulation period. For each manipulated stock, manipulators engage in fraudulent trading to create a fake image of an active market and attract the other investors to buy the stock. For example, manipulators control hundreds of accounts to purchase or sell a large amount of shares at the same time, influencing the stock price. Meanwhile, money transfers from (controlled) satellite accounts to a central account. During this process, the manipulator trade shares from one account to another without changing ownership and this fraudulent trading leads to an image of active market. In addition, other type of fraudulent trading behavior also exists in these manipulated stocks.

Network provides us a powerful mathematical tool to study complex interactions among different entities in financial systems^24^,^25^. Several efforts have been made to study stock market from the perspective of complex networks^26^,^27^,^28^,^29^,^30^,^31^,^32^. In these networks, nodes are companies, stocks, and even time series of stock prices. Links characterize certain correlation or affiliation among nodes. Recently, with the increasing availability of transaction data, researchers begin to study stock trading network which depicts the trading relationship among traders in stock markets^16^,^17^. Similar to complex networks from other fields, stock trading network also possesses a power-law degree distribution, a power-law strength distribution, and a power-law weight distribution^14^,^16^.

In stock trading network, nodes are traders involved in the transactions of a stock. Links represent the trading relationship among traders. For each transaction between two traders, there is a link pointing from the seller to the buyer. Generally, stock trading networks could be weighted, with the weights representing volume of transactions or frequency of transactions between traders. In this paper, we focus on the structural characteristics of stock trading network rather than the weight of links. Hence the studied stock trading network is a directed, unweighted network. Moreover, we use the daily trading network to refer to the trading network constructed according to the transactions occurred in a trading day.

Note that stock trading network is constructed from transaction data, which is generated by electronic trading system of stock exchange. As a result, stock trading network is closely related to the trading rules regulated by the stock exchange. Thus, before study the properties of stock trading network, we first briefly introduce the general trading rules in stock exchange. In stock market, a trader submits bid orders or ask orders to the electronic trading system when she wants to buy or sell shares. In electronic trading system, the list of bid orders is sorted in descending order of price and the list of ask orders is sorted in ascending order. For each of the lists of orders, orders with the same price are further sorted according to their submission time. Trading system matches a bid order and an ask order from the top of these two lists. Once a bid order is matched by an ask order, a transaction is executed generating a transaction entry in our data. If two matched orders do not have the same volume, the unexecuted part of the order with larger volume is taken as an unmatched order waiting for further match.

### Node roles in trading network

In this paper, we classify nodes into three categories according to their connectedness, measured with *z*-score 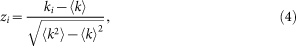
where *k_i_* is the degree of node *i* which is the sum of indegree and outdegree, 〈*k*〉 is the average degree of all nodes, 〈*k*^2^〉 is the second origin moment. According to previous research^14^,^16^,^17^, the degree distributions of the trading network follow power-law. The degree of most nodes are 1 and less than the average degree of the network, so the *z*-score of these nodes are less than 0. Meanwhile, a small proportion of nodes have large degree and the their *z*-score are greater. Based on our observations, nodes with *z*-score lager than 0.5 are taken as hubs, denoted by H. Nodes with *z*-score less than −0.05 are viewed as periphery nodes, denoted by P. The other nodes, with the *z*-score being between −0.05 and 0.5, are called connector nodes, denoted by C. The z-score thresholds are chosen based on our empirical results in Ref. 17. To be sure, it is a tricky to choose the z-score thresholds and we look forward to modeling of the trading behavior which could provide potential criteria for choosing z-score threshold. Figure 2 illustrates the three kinds of nodes in a trading sub-network, extracted from a daily trading network of a stock.

### Granger causality analysis

Granger causality test is a statistical hypothesis test for determining whether one time series is useful in forecasting another. According to Granger causality test, if a signal *X* exhibits a statistically significant correlation with a signal *Y*, then the past values of *X* should contain information that helps predict *Y* better than only leveraging the information contained in past values of *Y*. Here we determine whether the time series of link fraction are useful in forecasting the time series of stock price.

For time series of stock price, we consider the change of stock price between two consecutive days. We define the change of stock price in day *t* as 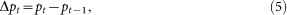
where *p_t_* is the closing price at day *t*. We use *X*^l^ to denote the time series of the fraction of links with type *l* (1 ≤ *l* ≤ 9). The null-hypothesis for our test is that *X*^l^ does Granger-cause the change of stock price. To test this null-hypothesis, we compare the following two regression equations 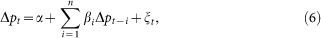

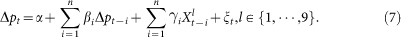


For the first linear regression model, *n* lagged values of Δ*p* are used to predict the change of stock price Δ*p_t_* at time t. For the second linear regression model, both the lagged values of Δ*p* and *X*^l^ are employed for prediction. The Granger causality analysis is conducted on 51 stocks over the whole year of 2004.

Granger causality test could provide some insight about which link type is potential at predicting stock price. However, Granger causality test is based on linear regression model and thus cannot uncover the relevant factors which are non-linearly predictive for stock price. To address this problem, we develop a three-layered feed forward neural network model which is non-linear model and could fully exploit the potential prediction power of its input.

To train the neural network, we divide all the data into two equal-sized parts: the training set and the test set. For the stocks in training set, the future stock price is used to train the neural network. For the stocks in test set, only the past time series of stock price and link types are known. To assess the contribution from link types, we compare the performance of neural networks with two different sets of inputs: (1) the time series of stock price and (2) the time series of stock price combined with one of the nine time series of links. The output of neural networks is the rate of price change, which is defined as: 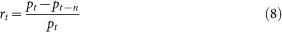
Furthermore, to test the predictive power of link types without Granger causality relation to stock price, we also combine the time series of these link types with the links types which Granger-cause the change of stock price.

The effective of prediction method is measured in terms of the Mean Absolute Percentage Error (MAPE) and the accuracy at predicting the rise or fall of stock price. MAPE is a measure to evaluate the accuracy of the predicted time series relative to the real time series, defined as: 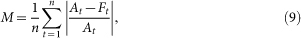
where *A_t_* is the real value and *F_t_* is the predicted value.

## Figures and Tables

**Table 1:** Results of Granger causality analysis (p-value < 0.05:**, p-value < 0.1:*)

*Lag day*	*X*^1^ *P − P*	*X*^2^ *P − C*	*X*^3^ *P − H*	*X*^4^ *C − P*	*X*^5^ *C − C*	*X*^6^ *C − H*	*X*^7^ *H − P*	*X*^8^ *H − C*	*X*^9^ *H − H*
1 day	0.170	0.481	0.302	0.246	0.781	0.035**	0.309	0.190	0.963
2 days	0.364	0.746	0.049**	0.518	0.412	0.088*	0.282	0.108	0.926
3 days	0.502	0.617	0.059*	0.568	0.620	0.084*	0.234	0.166	0.936
4 days	0.641	0.626	0.111	0.667	0.635	0.129	0.360	0.247	0.978
5 days	0.733	0.730	0.124	0.804	0.800	0.164	0.342	0.358	0.983
6 days	0.850	0.696	0.230	0.925	0.851	0.257	0.597	0.396	0.961
7 days	0.761	0.568	0.278	0.913	0.816	0.256	0.478	0.498	0.885

**Table 2:** Results of prediction (n = 3)

Lag	Evaluation	*I*_0_	*I*_3_	*I*_6_
2 days	MAPE(%)	6.29	4.92	6.25
	Accuracy(%)	51.7	52.5	49.2
3 days	MAPE(%)	4.69	4.69	4.70
	Accuracy(%)	57.5	56.7	55.0

**Table 3:** Results of prediction with both link type and historic stock price (n = 3)

Evaluation	*I*_0_	*I_p_*_,3_	*I_p_*_,3,1_	*I_p_*_,3,2_	*I_p_*_,3,4_	*I_p_*_,3,5_	*I_p_*_,3,6_	*I_p_*_,3,7_	*I_p_*_,3,8_	*I_p_*_,3,9_
MAPE(%)	4.69	4.50	**3.95**	4.52	4.80	4.56	4.84	4.83	4.39	4.61
Accuracy(%)	57.5	56.7	**61.7**	56.7	56.7	60.0	57.5	59.2	56.7	58.3

Evaluation	*I*_0_	*I_p_*_,6_	*I_p_*_,6,1_	*I_p_*_,6,2_	*I_p_*_,6,3_	*I_p_*_,6,4_	*I_p_*_,6,5_	*I_p_*_,6,7_	*I_p_*_,6,8_	*I_p_*_,6,9_
MAPE(%)	4.69	5.73	4.96	5.17	4.81	5.46	5.42	5.06	5.56	4.62
Accuracy(%)	57.5	55.0	55.8	55.8	57.5	55.0	55.8	55.8	57.5	56.7

**Table 4:** Results of prediction with only link types in trading network (n = 3)

Evaluation	*I*_3,1_	*I*_3,2_	*I*_3,4_	*I*_3,5_	*I*_3,6_	*I*_3,7_	*I*_3,8_	*I*_3,9_
MAPE(%)	**1.89**	1.98	2.39	2.21	2.02	1.94	1.95	2.07
Accuracy(%)	65.8	60.0	54.2	55.8	**69.2**	62.5	63.3	57.5

Evaluation	*I*_6,1_	*I*_6,2_	*I*_6,3_	*I*_6,4_	*I*_6,5_	*I*_6,7_	*I*_6,8_	*I*_6,9_
MAPE(%)	2.08	2.09	2.02	2.09	2.09	**1.86**	2.08	2.26
Accuracy(%)	52.5	53.3	**69.2**	55.0	55.0	**69.2**	57.5	54.2
